# Mechanical circulatory support in cardiogenic shock

**DOI:** 10.1186/s40560-023-00710-2

**Published:** 2023-12-19

**Authors:** Jun Nakata, Takeshi Yamamoto, Keita Saku, Yuki Ikeda, Takashi Unoki, Kuniya Asai

**Affiliations:** 1 Division of Cardiovascular Intensive Care, Nippon Medical School Hospital, 1-1-5 Sendagi, Bunkyo-Ku, Tokyo, 113-8603 Japan; 2 Department of Cardiovascular Dynamics, National Cerebral and Cardiovascular Center Research, Suita, Osaka Japan; 3 Department of Cardiovascular Medicine, Kitasato University, School of Medicine, Sagamihara, Kanagawa Japan; 4 Department of Cardiology and Intensive Care Unit, Saiseikai Kumamoto Hospital, Kumamoto, Japan

## Abstract

Cardiogenic shock is a complex and diverse pathological condition characterized by reduced myocardial contractility. The goal of treatment of cardiogenic shock is to improve abnormal hemodynamics and maintain adequate tissue perfusion in organs. If hypotension and insufficient tissue perfusion persist despite initial therapy, temporary mechanical circulatory support (t-MCS) should be initiated. This decade sees the beginning of a new era of cardiogenic shock management using t-MCS through the accumulated experience with use of intra-aortic balloon pump (IABP) and venoarterial extracorporeal membrane oxygenation (VA-ECMO), as well as new revolutionary devices or systems such as transvalvular axial flow pump (Impella) and a combination of VA-ECMO and Impella (ECPELLA) based on the knowledge of circulatory physiology. In this transitional period, we outline the approach to the management of cardiogenic shock by t-MCS. The management strategy involves carefully selecting one or a combination of the t-MCS devices, taking into account the characteristics of each device and the specific pathological condition. This selection is guided by monitoring of hemodynamics, classification of shock stage, risk stratification, and coordinated management by the multidisciplinary shock team.

## Epidemiology of cardiogenic shock

Cardiogenic shock is caused by the disease with impaired function of the myocardium, valve, conduction system, or pericardium, either in isolation or in combination. The most common pathology of cardiogenic shock is a clinical condition characterized by reduced myocardial contractility, which may lead to a vicious cycle of decreased cardiac output, low blood pressure, perpetuating further coronary ischemia and impaired contractility, ultimately resulting in multi-organ dysfunction. In addition, venous congestion leads to congestive organ damage, which is associated with increased mortality rate [1].

A contemporary registry has reported that as many as 81% of patients presenting with cardiogenic shock had underlying acute coronary syndrome (ACS) including acute myocardial infarction (AMI) [2]. An estimated up to 20% of patients with AMI potentially developed cardiogenic shock in the past, but recent clinical trials and observational studies have reported rates of  7–10% [3]. Although the development of cardiogenic shock has decreased over the years due to innovative therapies of percutaneous coronary intervention (PCI) for early revascularization and optimal medical therapy for AMI, the in-hospital mortality rate of AMI concomitant with cardiogenic shock (Killip IV) is still high. According to the Swiss AMI registry over the past 20 years, in-hospital mortality of AMI with cardiogenic shock (Killip IV) decreased from 62.2% in 1997 to 36.3% in 2017 [4]. Similar findings are also reported in Asian countries such as Japan. The Tokyo Cardiovascular Care Unit Network registry reported that in-hospital mortality rate of AMI with cardiogenic shock (Killip IV) ranged from 38.5% in 2007 to 27.2% in 2016 [5].

## Characteristics and hemodynamic effects of temporary mechanical circulatory support

The goal of treatment of cardiogenic shock is to improve abnormal hemodynamics and maintain adequate tissue perfusion in organs. If hypotension and insufficient tissue perfusion persist despite initial therapy, temporary mechanical circulatory support (t-MCS) should be initiated. Recently, the utilization rates of different modalities of t-MCS, such as intra-aortic balloon pump (IABP), venoarterial extracorporeal membrane oxygenation (VA-ECMO) and transvalvular axial flow pump (Impella; Abiomed Inc. Danvers, MA, USA) for the treatment of cardiogenic shock have changed remarkably. According to the Japanese Diagnosis Procedure Combination database containing 160,559 eligible patients over this decade, the prevalence of using IABP alone decreased significantly from 80.5% in 2010 to 65.3% in 2020 (P for trend < 0.001), whereas the prevalence of Impella alone increased significantly from 0.0% to 5.0% and ECMO from 19.5% to 29.6% (P for trend < 0.001 for both) [6]. In this transition period, it is necessary to select and use one or a combination of these devices appropriately based on the understanding of the characteristics of each device from the aspect of circulatory support and left ventricular (LV) unload, and tailored them to the pathological conditions of individual patients.

**Fig. 1:**
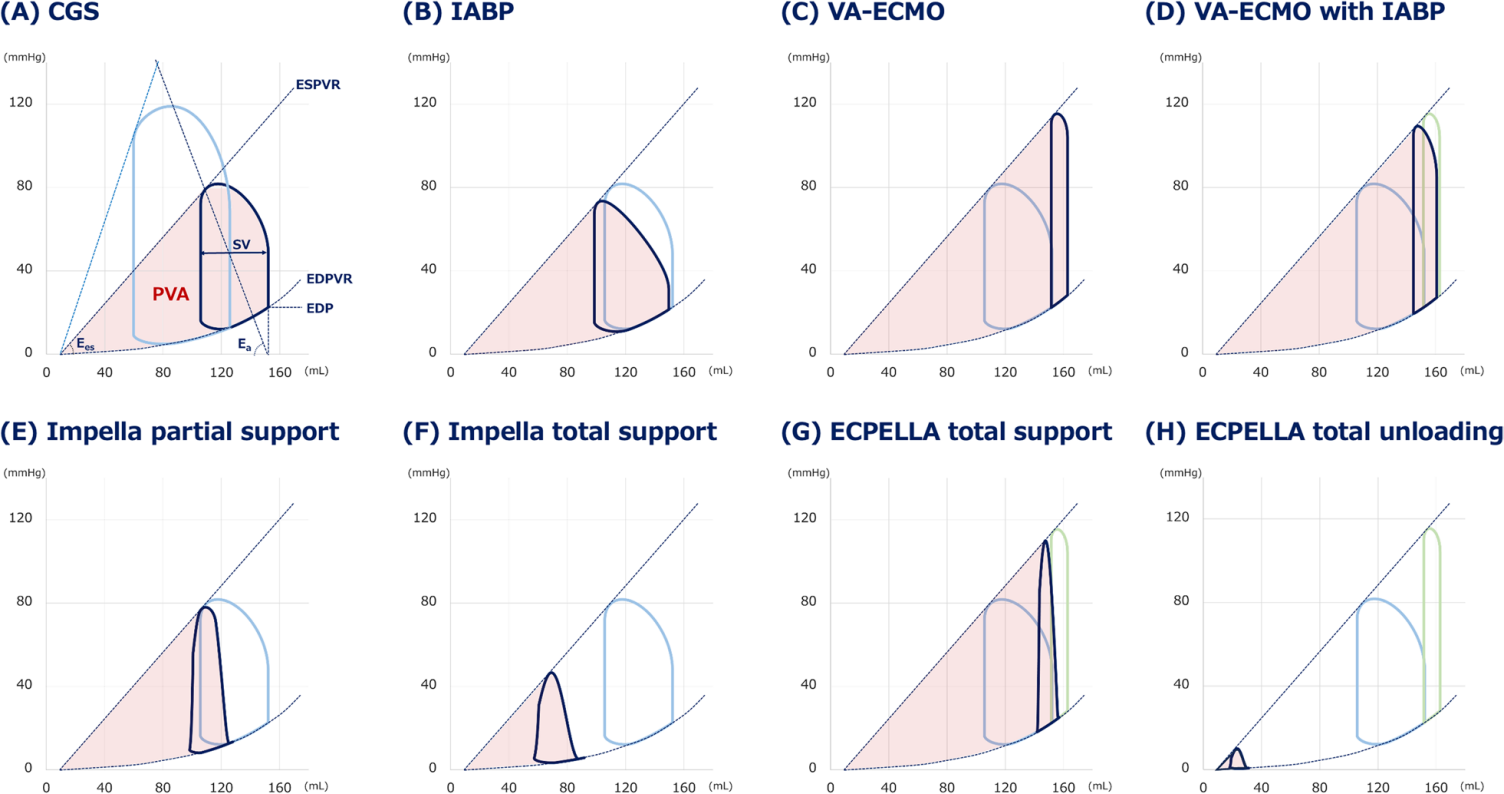
PV loops under various t-MCS conditions for the treatment of cardiogenic shock. Illustrations of PV loops in cardiogenic shock or cardiogenic shock with several MCS support conditions. **A** In cardiogenic shock, acute decrease of Ees shifts the PV loop rightward and reduces the stroke volume and LV pressure. **B**–**H** PV loops under various support conditions: IABP, VA-ECMO, VA-ECMO + IABP, Impella partial support, Impella total support, ECPELLA total support, and ECPELLA total unloading. *PV loop* pressure–volume loop, *t-MCS* temporary mechanical circulatory support, *CGS* cardiogenic shock, *IABP* intra-aortic balloon pump, *VA-ECMO* venoarterial extracorporeal membrane oxygenation, *ECPELLA* VA-ECMO + Impella, *Ea* effective arterial elastance, *Ees* end-systolic elastance, *SV* stroke volume, *PVA* pressure‒volume area, *EDP* end-diastolic pressure, *EDPVR* end-diastolic pressure‒volume relation, *ESPVR* end-systolic pressure‒volume relation

The mechanical effects on the LV varied among the systems used in t-MCS. The mechanical properties of the LV can be described by the pressure‒volume (PV) loop, with pressure and volume plotted on the same plane. The slope of the end-systolic pressure‒volume relation (ESPVR), E_es_, indicates the load-independent contractile function of the ventricle, while the end-diastolic pressure‒volume relation (EDPVR) represents the ventricular diastolic property. The systolic pressure‒volume area (PVA) (Fig. 1A) bounded by the ESPVR, EDPVR and the pulsatile systolic pressure‒volume curve of the PV loop, is the total mechanical energy generated by contraction and is linearly related to myocardial oxygen consumption [7, 8].

### IABP

IABP is still the most commonly used modality of t-MCS. The catheter itself is a double-lumen 7–8 Fr catheter with a polyethylene balloon with inflation and deflation synchronized with the electrocardiogram (ECG) or pressure triggers. IABP increases diastolic blood pressure, decreases afterload, decreases myocardial oxygen consumption, increases coronary artery perfusion and cardiac output, and provides modest ventricular unloading. Monitoring for proper IABP operation checks that the aortic pressure waveform obtained from the IABP balloon tip is appropriately augmented in diastole and unloaded in systole.

The direct effect of IABP on the LV is a decrease in end-systolic pressure due to rapid deflation of the balloon during systole, resulting in systolic unloading. IABP-induced systolic unloading increases stroke volume (SV) **(**Fig. 1B) to a limited extent [9, 10]. Although there is no direct mechanical effect on the LV during diastole, the preservation of LV function by maintaining or increasing coronary blood flow and maintenance of peripheral organ perfusion by increasing pulse pressure are important clinical benefits of IABP.

Regarding the prognostic value of IABP in increasing coronary artery perfusion pressure, the BCIS-1 trial reported that in high-risk PCI with ejection fraction (EF) < 30%, IABP support reduced major adverse cardiac and cerebrovascular events at discharge and decreased mortality by 34% at a median of 51 months [11]. There is no clear evidence that IABP improves the prognosis of AMI, although it has a hemodynamic stabilizing effect by increasing coronary perfusion pressure. The CRISP trial showed that IABP during primary PCI did not reduce infarct size in anterior ST-segment elevation myocardial infarction (STEMI) [12]. Although IABP is widely used clinically, a prospective randomized controlled trial failed to demonstrate conclusive proof of its benefits for cardiogenic shock. The IABP-Shock II study published in 2013 showed that IABP placement in patients with cardiogenic shock due to ACS did not reduce 30-day mortality and showed no improvement in 6-year outcomes [13].

Based on these findings, the 2014 European Society of Cardiology (ESC) guidelines downgraded the use of IABP in patients with cardiogenic shock due to ACS from “recommended” (Class I) in the past to “not recommended” (Class III) [14]. On the other hand, the Japan Circulation Society guidelines recommend IABP for mechanical complications of AMI (Class I), recommend IABP to be considered in patients with cardiogenic shock and prolonged myocardial ischemia after reperfusion (Class IIa), and do not recommend IABP for routine use in patients with cardiogenic shock (Class III) [15, 16].

Given the paucity of sufficiently powered randomized trials of t-MCS, most data on complication rates of IABP originate from observational case series and registries. Kapur et al. [17] reviewed evidence from available randomized trials and compared the complication rates obtained from randomized trials with case series and comparative observational studies. In their large sample, bleeding occurred in 12.9% of patients treated with IABP, 27.7% treated with Impella, and 28.2% treated with VA-ECMO, while limb ischemia occurred in 1.5%, 4.2%, and 14.3%, respectively, and stroke in 3.1%, 4.9%, and 8.2%, respectively. The apparent trend from these data showed higher rates of bleeding and vascular injury with Impella compared to IABP, which are associated with devices requiring larger bore access, and higher rate of stroke also with Impella than with IABP. All three rates were higher still with VA-ECMO. Thus, given the lack of conclusive proof of benefits of IABP for cardiogenic shock but an apparently favorable safety profile, the use of IABP should be considered when other t-MCS devices are not available, or when the risk of complications including stroke, bleeding, and limb ischemia is increased by management using other t-MCS devices.

### VA-ECMO

VA-ECMO is a percutaneous ventricular assist device consisting of a membrane artificial lung and a closed circuit with a centrifugal pump. A venous cannula (17–24 Fr) drains deoxygenated blood into a membrane oxygenator for gas exchange, and oxygenated blood is subsequently infused into the patient via an arterial cannula (14–19 Fr). Pressure generated by VA-ECMO, which is the product of VA-ECMO flow and systemic vascular resistance, loads the LV during systole. Although the venous return to the LV is reduced by VA-ECMO, the increase of LV afterload reduces SV. As a result, especially in a depressed LV, VA-ECMO enlarges PVA (Fig. 1C) [18, 19]. Therefore, IABP or Impella is used to reduce the LV afterload induced by VA-ECMO in clinical practice. However, as shown in Fig. 1D, IABP provides limited leftward shift of the PV loop under the condition of VA-ECMO support.

Because VA-ECMO provides the highest flow rate (3.0–7.0 L/min) among all the t-MCS devices, which improves metabolic derangement and deleterious systemic effects, it may confer a modest mortality benefit in cardiogenic shock [20, 21]. In the SAVE-J trial, Sakamoto et al. [22] examined the beneficial effect of VA-ECMO as resuscitation therapy in additional to conventional cardiopulmonary resuscitation (CPR) for cardiac arrest patients.

The International Society for Heart and Lung Transplantation/Heart Failure Society of America guidelines state that VA-ECMO may be considered as an adjunct to conventional CPR for extracorporeal cardiopulmonary resuscitation (Class IIc) in patients with cardiac arrest, who are expected to regain cardiac function and for whom appropriate CPR is being performed [23]. ESC guidelines also state that the use of VA-ECMO may be considered for severe cardiogenic shock, in-hospital and out-of-hospital cardiac arrest in individual cases (Class IIb) [14]. In Japan, VA-ECMO is considered for use in patients with cardiogenic shock refractory to drug therapy (Class IIa), and may also be considered in situations of progressive circulatory failure due to mechanical complications or in situations where maintenance of circulation until surgery is difficult (Class IIb) [15, 16].

Although VA-ECMO has established an indispensable position in resuscitation management, notably the disadvantages of this therapy need to be considered, such as bleeding, thromboembolic events, and hemolysis. In addition, considerable attention in management is required to avoid the risk of increased myocardial oxygen demand and myocardial remodeling due to increased LV afterload caused by retrograde blood flow as well as exacerbation of oxygenation.

In the setting of concomitant respiratory failure, after the LV recovers and begins to eject, deoxygenated blood returned from the compromised lungs will begin to merge with oxygenated blood from the ECMO circuit at a site known as the mixing point. Resulting regional or differential hypoxemia can then occur, and the more distal the mixing point, the greater the risk for cerebral hypoxemia. It is recommended that the right radial artery as well as right upper torso pulse oximetry should routinely be used for blood gas as well as tissue oxygenation to immediately detect regional or differential hypoxia [24].

### Impella

Impella is a non-pulsatile axial flow Archimedes-screw pump designed to pump blood from the LV into the ascending aorta. The 12 Fr (Impella 2.5) and 14 Fr (Impella CP) devices that provide maximal flow rates of 2.5 L/min and 3.7 L/min, respectively, are designed to be placed via the femoral artery. On the other hand, the 21 Fr (Impella 5.5) device with maximal flow rate of 5.5 L/min requires a surgical cutdown for deployment axillary or femoral artery. A possible advantage of the axillary approach is the potential for long-term support. Concerning the hemodynamic effects, Impella reduces myocardial oxygen consumption, improves mean arterial pressure, and reduces pulmonary artery wedge pressure (PAWP), which reduces native LV stroke work and wall stress.

Impella drains blood from the LV and pumps it into the aorta. Regarding monitoring of pump flow settings, attention should be paid to pulse pressure and mean arterial pressure. A non-pulsatile arterial signal suggests uncoupling between LV and systemic pressure. A change from pulsatility to nonpulsatility should trigger echocardiography to ensure proper positioning of the device, but nonpulsatility may suggest that the left ventricle is well unloaded [25].

We have reported the impact of Impella on the PV loop in a canine study and a clinical study in patients with AMI complicated with cardiogenic shock [26, 27]. With Impella partial support, in which the LV remains ejecting, total cardiac output increases thereby increasing blood pressure. This leads to increase in LV end-systolic volume despite marked reduction in LV end-diastolic volume. Consequently, partial support by Impella does not sufficiently reduce PVA (Fig. 1E). On the other hand, with Impella total support, LV no longer ejects because LV pressure never reaches blood pressure. Thus, total support with Impella renders PVA extremely small (Fig. 1F).

The safety and efficacy of using Impella in AMI with cardiogenic shock has been verified in clinical trials. The USpella Registry showed hemodynamic improvement of using Impella 2.5 in AMI patients with cardiogenic shock who did not improve with inotropic drugs or IABP [28]. In the MACH II study that examined the effect of Impella 2.5 on long-term cardiac function in patients with anterior STEMI, significant improvement was observed after three years when compared to a control group treated with conventional therapy [29]. Ikeda et al. [30] reported the treatment outcome of Impella for AMI (Killip IV) in the J-PVAD registry, showing favorable 30-day survival rate of 80.9% with Impella alone compared with 63.1% overall. Concerning the effect of Impella support with revascularization and optimal medical therapy (OMT) on cardiac function, mean (± SD) left ventricular ejection fraction (LVEF) was 35 ± 12.1% prior to Impella treatment and improved significantly to 44.7 ± 11.0% at Impella explant (*P* < 0.001).

Two randomized control trials compared IABP with Impella. In the ISAR-SHOCK study reported in 2008, 25 patients with AMI and cardiogenic shock were randomized to treatment with Impella 2.5 (12 patients) or IABP (13 patients). The primary endpoint of cardiac output (CO) improved significantly in the Impella group compared with the IABP group (0.49 ± 0.46 vs. 0.11 ± 0.31, *P* < 0.05) [31]. In contrast, the IMPRESS trial reported in 2015 found no difference in survival outcome between Impella and IABP. This trial randomized 48 patients with severe cardiogenic shock caused by AMI to Impella CP (24 patients) or IABP (24 patients). The primary endpoint of all-cause mortality at 5 years was 11/24 (46%) in the Impella CP group and 12/24 (50%) in the IABP group, with no significant difference between the two groups [32].

Although Impella has significant benefits of hemodynamic stabilization and LV unloading, its use has potential risk of complications. The most common complications of Impella are limb ischemia, bleeding requiring blood transfusion, and hemolysis. These complications require accurate evaluation and preventive measures, which should be detailed in the protocol to ensure safe and reliable management for better therapeutic results.

### ECPELLA

In patients with prolonged shock and cardiac arrest, MCS using a combination of VA-ECMO and Impella, so-called ECPELLA or ECMELLA, is recently used to maintain organ perfusion and oxygenation and restore cardiac function by LV unloading.

**Fig. 2:**
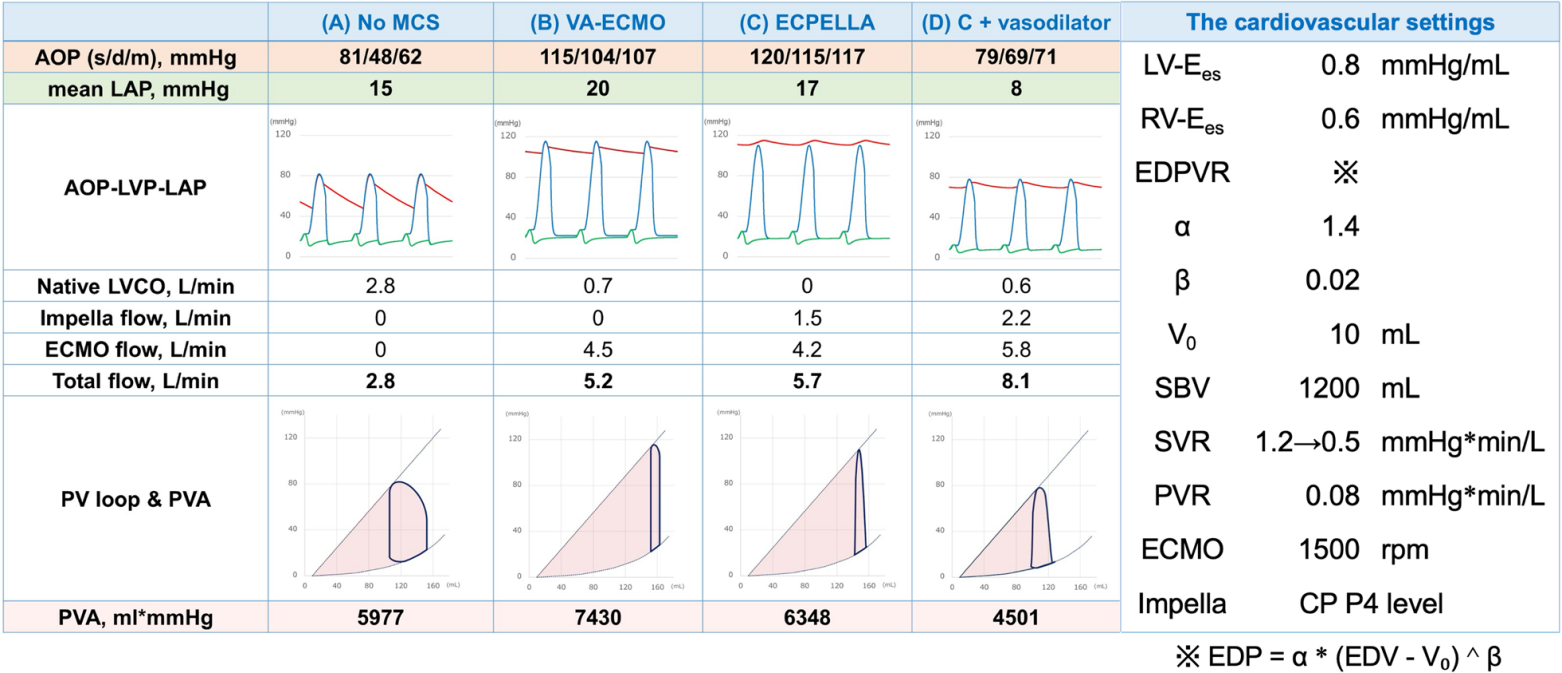
The impact of aortic pressure on ECPELLA-supported PV loop. In cardiogenic shock condition (**A**), VA-ECMO markedly increases total systemic flow, while loading LV and increasing PVA (**B**). Addition of Impella to VA-ECMO decreases PVA (**C**). Further addition of a vasodilator that decreases blood pressure further reduces PVA markedly, and further increases total systemic flow (**D**). The simulation was conducted using our electrical model reported previously [33]. The cardiovascular parameters employed for the simulation are depicted in the right column. *MCS* mechanical circulatory support, *VA-ECMO* venoarterial extracorporeal membrane oxygenation, *ECPELLA* combination of VA-ECMO and Impella, *AOP* aortic pressure, *LAP* left atrial pressure, *LVP* left ventricular pressure, *LVCO* left ventricular cardiac output, *PV* pressure‒volume, *PVA* pressure‒volume area, *LV-E*_*es*_ left ventricular end-systolic elastance, *RV-E*_*es*_ right ventricular end-systolic elastance, *EDPVR* end-diastolic pressure–volume relationship, *V*_*0*_ the volume intercept of end-systolic pressure volume relationship of LV, *SBV* stressed blood volume, *SVR* systemic vascular resistance, *PVR* pulmonary vascular resistance

As mentioned above, although VA-ECMO has the advantage of increasing blood pressure, it may potentially increase LV afterload and shift the PV loop to the right, leading to an increase in PVA. ECPELLA reduces the VA-ECMO-induced increase in PVA, while blood pressure continues to increase, as shown in simulation studies [33] (Fig. 1G). Thus, ECPELLA is a treatment that allows hemodynamic stabilization while reducing PVA. The degree of PVA reduction varies depending on the ECPELLA support condition. In addition, we often experience the “total unloading” condition that represents a marked reduction of PVA (Fig. 1H) during ECPELLA management [34, 35]. In the management of PVA, control of blood pressure is critical not only in terms of reducing PVA, but also reducing overload to the pump head of the centrifugal and axial pumps such as VA-ECMO and Impella. Figure 2 illustrates the hemodynamic and PV loop alterations under various ECPELLA settings. The cardiovascular parameters employed for the simulation are depicted in the right column. In the simulation, we adjusted the right and left ventricular systolic function (Ees) and diastolic function (α, β), the *x*-intercept of ESPVR, stressed blood volume (SBV), pulmonary (PVR) and systemic (SVR) vascular resistance to create the cardiogenic shock condition as shown in Fig. 2A. In the simulation, we sequentially established ECPELLA by incorporating VA-ECMO (Fig. 2B) and Impella CP (P4 level) (Fig. 2C), maintaining the initial cardiovascular settings. When vasodilators are further administered to decrease systemic vascular resistance (1.2–0.5 mmHg*min/L), the PV loop changes as shown in Fig. 2D. Compared to ECPELLA total support (Fig. 2C), ECPELLA with blood pressure lowering showed remarkable reduction of PVA and increase of total flow, despite the partial support condition.

Although ECPELLA reduces PVA remarkably by effectively regulating the flow rate between the VA-ECMO and Impella, reducing PVA to the limit is tantamount to collapsing the LV. In some cases, LV suction by Impella may have the risk of causing ventricular arrhythmias, hemolysis, and pump malfunction. In addition, optimal management of blood pressure in patients treated with ECPELLA is also important, since excessively elevated mean blood pressure may be a paroxysmal cause of intracranial hemorrhage. Note that aortic regurgitation (AR) may occur in the gap between the Impella shaft and the aortic valve leaflets, which would hamper PVA reduction by Impella [36]. Thus, appropriate intravascular volume and optimal blood pressure are indispensable for the control of t-MCS flow and prevention of AR.

Currently, controversy exists regarding optimal regulation of PVA when the heart is recovering from acute injury. Mazurek et al. [37] reported that acute reloading induces cardiac apoptosis. On the other hand, Diakos et al. [38] showed that cardiac reloading through an LV assist device normalizes metabolism. Further studies are needed to clarify the optimal PVA regulation by t-MCS according to the clinical situation.

In a clinical study, Schrage et al. [39] investigated the efficacy of ECMELLA in patients with cardiogenic shock by comparing 255 patients treated with VA-ECMO alone and 255 patients with ECMELLA (VA-ECMO + Impella). They reported significantly improved 30-day mortality in the ECMELLA group compared to the VA-ECMO group (HR 0.79, 95% confidence interval [CI] 0.63‒0.98, *p* = 0.03). Furthermore, a sub-analysis showed improved 30-day mortality in the group receiving Impella within 2 h of VA-ECMO initiation (HR 0.76, 95% CI 0.60‒0.97, *p* = 0.03), but not in the group receiving Impella more than 2 h later (HR 0.77, 95% CI 0.51‒1.16, *p* = 0.22). Regarding the effect of LV unloading in patients with profound cardiogenic shock caused by AMI treated with ECPELLA, a report from the J-PVAD registry studied 300 patients with a high rate of out-of-hospital cardiac attack (32.3%), long duration of shock-to-Impella support [176 (103‒319) min] and high lactate level [8.7 (4.3‒13.4) mmol/L]. Of the patients treated with ECPELLA, 50 patients with available LVEF data prior to Impella support and at Impella explant were analyzed. Mean LVEF in these patients improved from 24.9 ± 14.2% prior to treatment to 44.0 ± 16.6 at Impella explant (*P* < 0.001) [30]. Despite improvement in short-term mortality, patients treated with ECPELLA had significantly higher rates of bleeding, hemolysis, limb ischemic, and renal replacement therapy compared to those treated with VA-ECMO alone. Cappannoli et al. [40] conducted a systematic literature review comparing ECPELLA with VA-ECMO alone in patients with cardiogenic shock. ECPELLA was associated with increased bleeding (risk ratio [RR] 1.45, 95% CI 1.20–1.75), hemolysis (RR 1.71, 95% CI 1.41–2.07), limb ischemia (RR 1.43, 95% CI 1.17–1.75), need for kidney replacement therapy (RR 1.54, 95% CI 1.19–1.99), and a non-significant increase in severe infections (RR 1.26, 95% CI 0.84–1.89) compared with VA-ECMO alone.

## Monitoring for the management of t-MCS

The intensity and degree of invasiveness of monitoring should depend on the severity and degree of instability of shock, the underlying etiology, the comorbidities, and the patient’s metabolic profile. Basic monitoring is advised to include serial assessment of lactate from blood gas analyses and monitoring of mixed venous or central venous blood saturation (SVO_2_) and urine output. Patients supported by t-MCS should be monitored with an arterial line, a central venous catheter, regular imaging by transthoracic echocardiogram and frequent use of pulmonary artery catheters [41].

Changes in both systemic and pulmonary arterial pulse pressure, or pulsatility, reflect the native heart recovery. Absent or very low pulsatility suggests severe cardiac contractile dysfunction or markedly reduced ventricular ejection. On the other hand, increasing pulsatility may be indicative of a recovering heart and improved native heart function. ETCO_2_ concentration can also be a useful parameter for detecting native heart recovery [42].

Daily echocardiography is advised to evaluate LV and right ventricular (RV) volume, recovery of native heart functions, signs of aortic or mitral valve regurgitation, pericardial effusion, LV thrombus, and the correct placement of the device.

Early placement of pulmonary artery catheterization (PAC) is advised to guide device settings, identify the potential need for escalation and venting, and evaluate signs of myocardial recovery. The American Heart Association scientific statement suggests placement of pulmonary artery catheter (PAC) to guide t-MCS device selection and use, unless there are absolute contraindications. Continuous PAC assessments combined with noninvasive imaging may also facilitate the appropriate escalation of t-MCS if clinical improvement is not observed with an initial t-MCS platform. Pressure tracings can be monitored continuously, and cardiac output measurements can be performed every 1–2 h, depending on shock severity [43].

In practice, PACs are used with the aim to identify the etiology of cardiogenic shock. For example, LV-dominant congestion in cardiogenic shock is often characterized by an elevated PAWP or LV end-diastolic pressure > 15 mmHg. Cardiac power output (CPO) can be obtained by a simple calculation: multiplying mean arterial pressure by cardiac output and dividing by a constant of 451. CPO has been shown to be the strongest hemodynamic predictor of mortality in the SHOCK trial and other clinical study [44–46]. Right ventricle (RV)-dominant congestion in cardiogenic shock is accompanied by a relatively normal PAWP in the setting of elevated right atrial (RA) pressure (> 15 mmHg) and elevated RA pressure to PAWP ratio (> 0.63 or 0.86), which should prompt further evaluation for RV failure [47–49]. In addition, the pulmonary artery pulsatility index [PAPi = (systolic pulmonary artery pressure − diastolic pulmonary artery pressure)/RA] is a hemodynamic variable used to identify RV failure. The prognostic cutoff value of PAPi differs between patients with AMI-cardiogenic shock (≤ 0.9) and patients with heart failure undergoing left ventricular assist device implantation (< 1.85) [50–52]. However, as the aforementioned hemodynamic parameters derived from PAC may be confounded by volume resuscitation, passive congestion, or concurrent valvular disease, integrating the assessment results of these invasive hemodynamic parameters with noninvasive cardiac imaging (echocardiography) is needed for diagnosing RV or biventricular failure.

## Selection and escalation of t-MCS for patients with cardiogenic shock

The goal of cardiogenic shock treatment using t-MCS is to maintain adequate tissue perfusion and to break the vicious cycle of hemodynamic abnormalities. In the acute heart failure setting, Shiraishi et al. [53] advocated the importance of time-sensitive approach in the management of cardiogenic shock, including adjunctive t-MCS. When there are signs of tissue hypoperfusion including hemodynamic hypotension findings such as systolic BP (sBP) < 90 mmHg or mean BP (mBP) < 65 mmHg together with lactate level > 2 mmol/L, initial management should include administration of vasoactive agents, inotropic drugs and volume loading to improve sBP to > 90 mmHg or mBP > 65 mmHg with improved tissue perfusion. If another evaluation conducted within 10 min of the first evaluation and treatment reveals persistent hypoperfusion and shock signs, consideration of initiation of t-MCS is recommended.

**Fig. 3:**
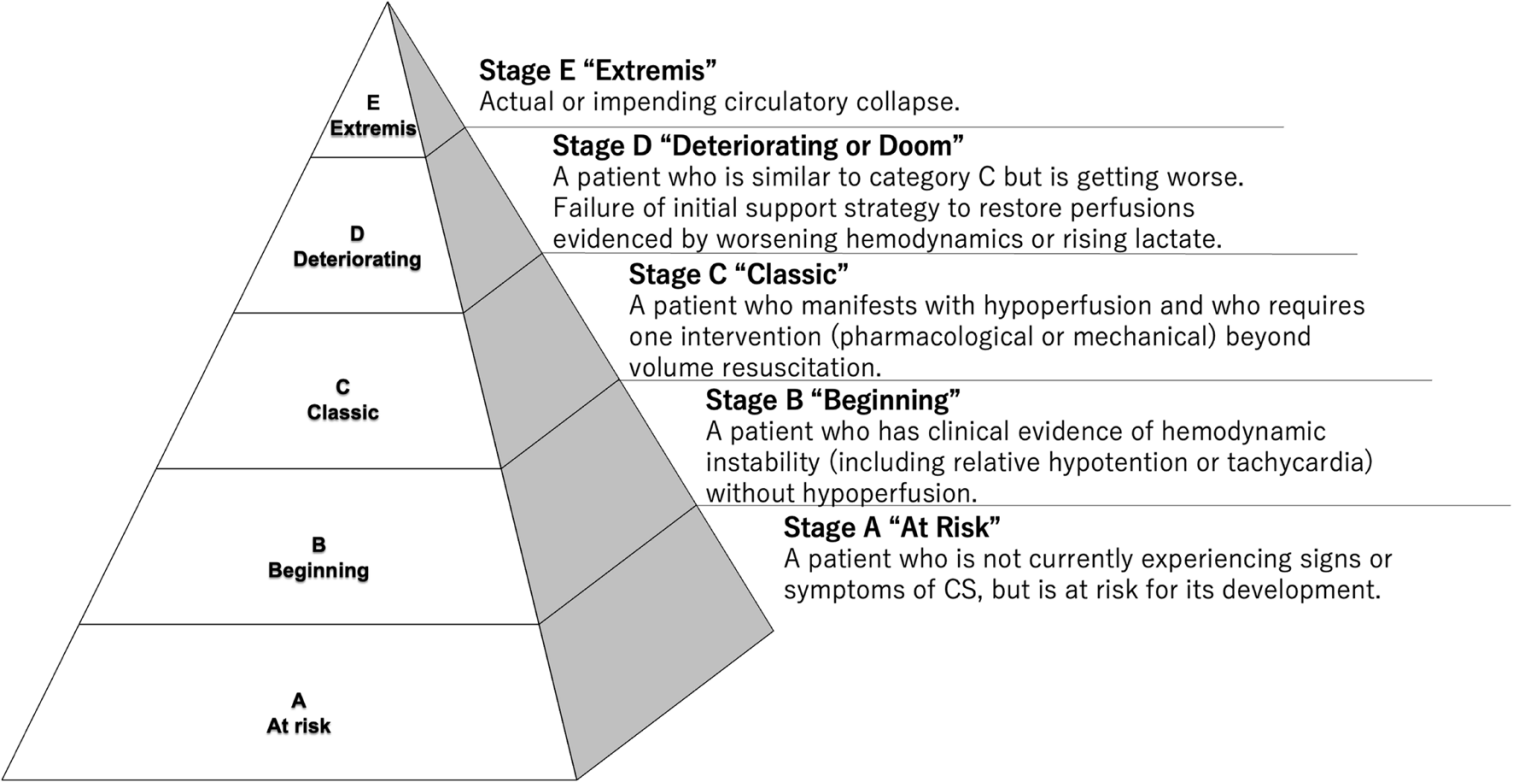
SCAI cardiogenic shock-classification. This figure was modified from Fig. 4 of the article. “SCAI SHOCK Stage Classification Expert Consensus Update: A Review and Incorporation of Validation Studies: This statement was endorsed by the American College of Cardiology (ACC), American College of Emergency Physicians (ACEP), American Heart Association (AHA), European Society of Cardiology (ESC) Association for Acute Cardiovascular Care (ACVC), International Society for Heart and Lung Transplantation (ISHLT), Society of Critical Care Medicine (SCCM), and Society of Thoracic Surgeons (STS) in December 2021 [56]

In 2019, American Society for Cardiovascular Interventions (SCAI) experts published a consensus statement on the classification of cardiogenic shock [54]. The SCAI classification can be used easily at the bedside. It stratifies patients with cardiogenic shock into 5 categories: stage A is at risk; stage B is beginning of shock; stage C is classic cardiogenic shock; stage D is deteriorating or doom; and stage E is extremis (Fig. 3) [55]. By design, the SCAI classification system accounts for changes in clinical trajectory, allows more granularity in patient description, is specifically designed for this patient population, and can be used to optimize patient selection for future cardiogenic shock trial enrollment [56].

Jentzer et al. [57] reviewed a database of 10,004 cardiogenic shock patients admitted to the cardiac intensive care unit at the Mayo Clinic between 2007 and 2015. In this cohort, they staged cardiogenic shock using the SCAI shock classification system and assessed the outcomes. The in-hospital mortality rates were 3.0% for stage A, 7.1% for stage B, 12.4% for stage C, 40.4% for stage D, and 67.0% for stage E, demonstrating the accuracy of the SCAI stage classification in stratifying clinical outcome. Hanson et al. [58] studied 300 AMI-cardiogenic shock patients enrolled in the NCSI registry between 2016 and 2019 and reported the prognostic value of the SCAI cardiogenic shock staging when applied at admission and at 24 h after initiation of t-MCS and completion of PCI. Among patients classified as stage C at admission, patients without worsening of stage after 24 h had survival rate of 84%, whereas patients who worsened from stage C to stage D had survival rate of 55%, and those from stage C to stage E had low survival of 17%. These results suggest that the key to improving survival outcome is to assess patients with cardiogenic shock for progression of the condition over time, to evaluate the stage of cardiogenic shock according to severity, and to introduce t-MCS at the appropriate timing.

**Fig. 4:**
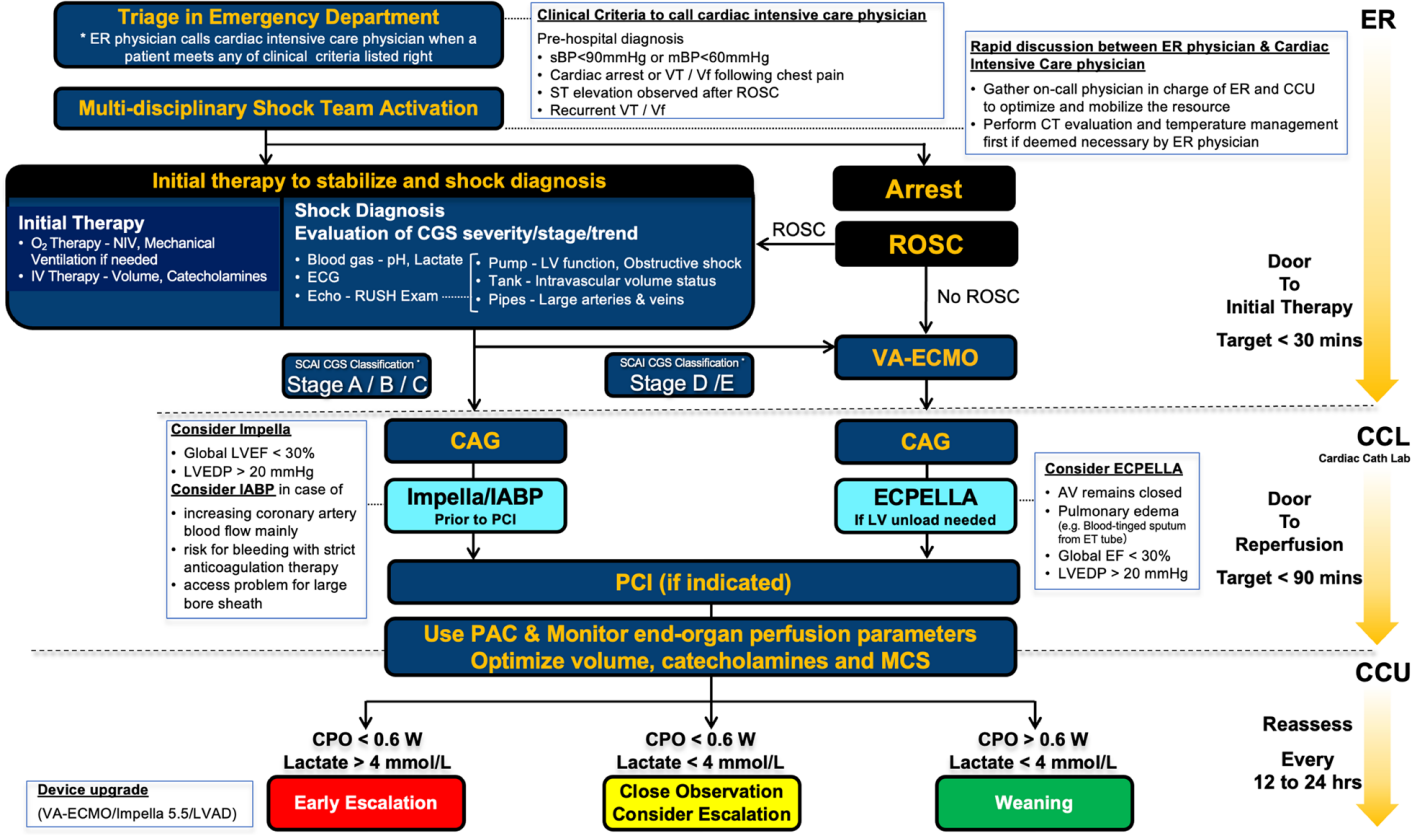
Protocol of selection and escalation of temporary mechanical circulatory support. *CAG* cardiogenic shock, *IABP* intra-aortic balloon pumping, *ROSC* return of spontaneous circulation, *VA-ECMO* venoarterial extracorporeal membrane oxygenation, *ECPELLA* combination of VA-ECMO and Impella, *LVEF* left ventricular ejection fraction, *LVEDP* left ventricular end-diastolic pressure, *CPO* Cardiac power output, *PCI* percutaneous coronary intervention, *PAC* pulmonary artery catheter, *VT* ventricular tachycardia, *Vf* ventricular fibrillation, *sBP* systolic blood pressure, *mBP* mean blood pressure, *CCU* cardiovascular care unit

Currently, the standardized and effective strategy for initiation and management of t-MCS in patients with cardiogenic shock includes timely assessment and tailored interventions prior to the development of the aforementioned detrimental cascade of shock status exemplified by the SCAI cardiogenic shock classification. A proposed management protocol for selection and escalation of t-MCS is presented in Fig. 4.

Initially, triage of a patient with shock is performed in ER based on pre-hospital information. Then, initial oxygenation therapy and IV therapy are started, together with diagnosis of cardiogenic shock by blood gas, ECG, and Echo (RUSH exam), and evaluation of shock severity and trend. Cardiogenic shock is staged based on SCAI shock classification. For Stage A to C of the SCAI shock classification, volume infusion and intravenous inotropic agents are initiated when there are signs of hypoperfusion such as peripheral coldness, oliguria, and elevation of lactate, along with hypotension findings such as persistent low blood pressure (sBP < 90 mmHg); or intravenous inotropic agents are given to maintain end organ perfusion when blood pressure is normal (sBP > 90 mmHg). Then, if findings of hypoperfusion persist with global LVEF < 30% and increase of LV end-diastolic pressure to > 20 mmHg, initiation of Impella is considered for improvement of hemodynamics. However, initiation of IABP instead of Impella should be considered if the purpose of support is mainly to increase coronary artery blood flow without LV unloading, in patients on strict anticoagulation therapy with high risk of bleeding, or in patients with problem of access for large bore sheaths. For Stage D or E, such as refractory cardiogenic shock with ventricular tachycardia or ventricular fibrillation or cardiac arrest requiring CPR, VA-ECMO is the first-line t-MCS to stabilize hemodynamics and improve end organ hypoperfusion. Moreover, in patients with worsening of pulmonary congestion or insufficient aortic valve opening caused by LV afterload from using VA-ECMO, additional LV unloading using Impella or IABP should be considered. After initiation of appropriate t-MCS device, early revascularization with PCI for ACS or ischemic heart disease is performed if indicated, with a target door-to-reperfusion time < 90 min. Finally, before leaving the catheter laboratory, PAC is placed to monitor hemodynamics with the purpose to optimize the volume, catecholamine, and t-MCS using clinical and hemodynamic variables to assist end-organ perfusion.

The management of t-MCS for patients with cardiogenic shock in cardiac care unit (CCU) is primarily based on a comprehensive assessment of clinical, laboratory, imaging, and hemodynamic parameters [43]. Contrary to echocardiogram that has the limitations of providing information for only a single point in time and high susceptibility to interobserver bias, PAC has the ability to monitor serial changes over time and is free from interobserver bias.

Based on evidence on reliable and useful variables for predicting outcome of cardiogenic shock, stratification of patients according to CPO (> or < 0.6 W) and lactate measurements (> or < 4 mg/dL) within 12–24 h is recommended to guide clinical decision such as early device escalation, close observation, or weaning catecholamine and t-MCS [59].

## Weaning of t-MCS

**Fig. 5:**
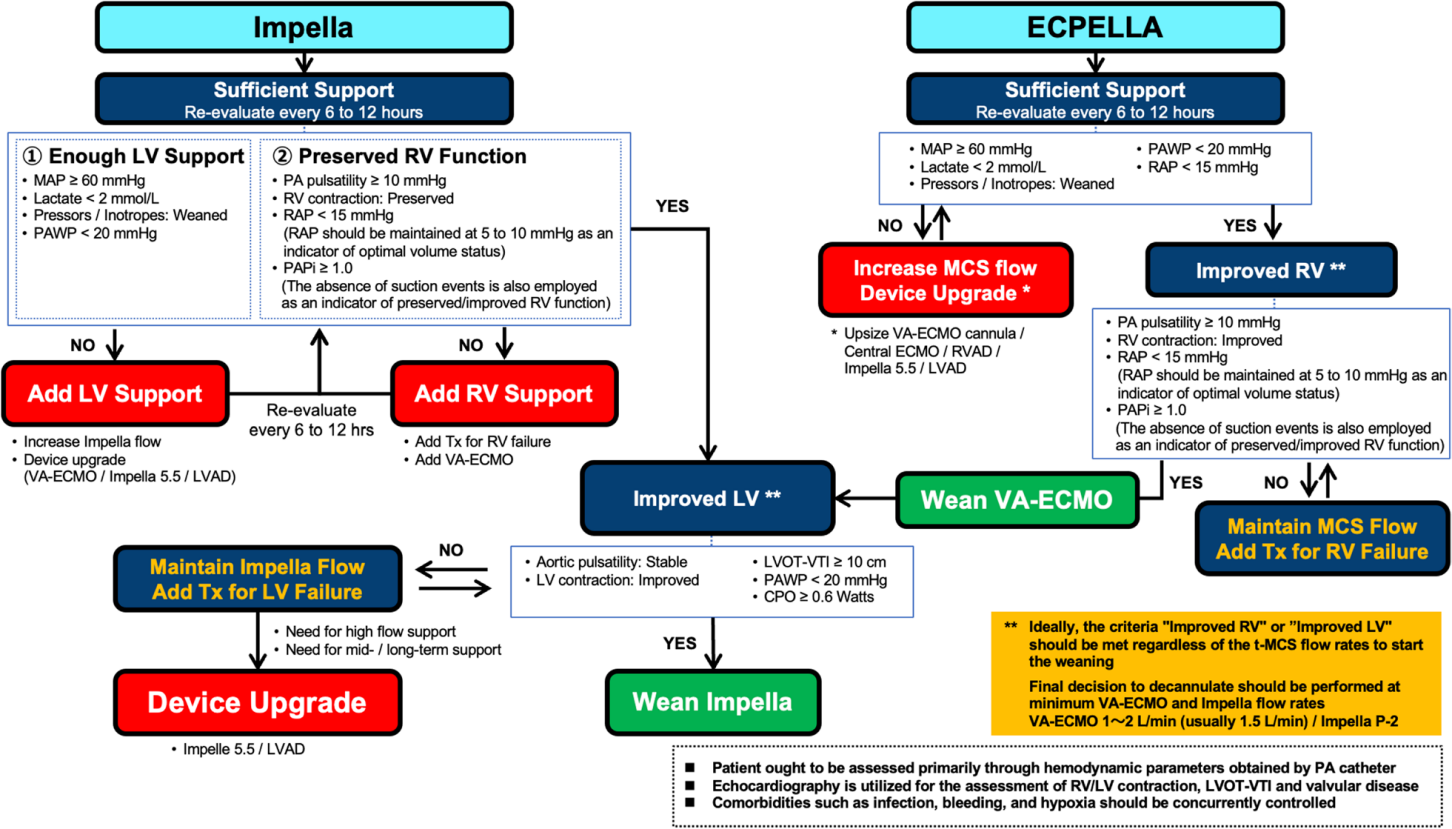
Protocol of weaning of temporary mechanical circulatory support. *MCS* mechanical circulatory support, *VA-ECMO* venoarterial extracorporeal membrane oxygenation, *ECPELLA* combination of VA-ECMO and Impella, *LV* left ventricular, *MAP* mean arterial pressure, *PAWP* pulmonary artery wedge pressure, *PA* pulmonary artery, *RV* right ventricular, *LV* left ventricular, *RAP* right atrial pressure, *PAPi* pulmonary artery pulsatility index, *VAD* ventricular assist device, *LVOT-VTI* left ventricular outflow tract velocity time integral, *CPO* cardiac power output

Individual patients have different etiologies and phenotypes of heart failure, and the clinical course after t-MCS induction varies. Improvements in blood pressure, serum lactate level, and end-organ function are indicators of adequate t-MCS support, but are not indicators of cardiac recovery. Clinical parameters that indicate improvement of cardiac performance should be used as weaning criteria. During the t-MCS weaning process, focusing on pulse pressure is essential. PAPi calculated using pulmonary artery pulse pressure is one of the indices used to evaluate RV function [60]. Initiation of t-MCS often leads to the disappearance of pulse pressure, and its reappearance indicates ameliorated cardiac performance. Maintaining adequate t-MCS flow and waiting for spontaneous reappearance of pulse pressure before proceeding to weaning are crucial. Prompt and meticulous attention is indispensable subsequent to the removal of t-MCS devices, because sudden hemodynamic changes may occur. Decannulation of t-MCS device often requires surgical suturing, and recannulation presents challenges. One of the most prudent processes of managing patients undergoing t-MCS is weaning. Therefore, well-defined criteria for deciding to proceed with t-MCS weaning ought to be established. A proposed management protocol for weaning from Impella or ECPELLA is presented in Fig. 5.

In patients on ECPELLA support, VA-ECMO weaning is usually done first, followed by Impella weaning. The Impella or ECPELLA weaning process entails three steps: first, assessment of whether support is sufficient; second, assessment of right ventricular function; and third, assessment of left ventricular function. In the first step, improvement of end-organ perfusion and intracardiac pressure should be evaluated. Elevated blood pressure, normalization of serum lactate [61], and weaning of pressors/inotropes are valuable indicators of improved end-organ perfusion . Amelioration of intracardiac pressure, which is evaluated using right atrial pressure and PAWP, is also essential. The second step requires meeting the criteria of right atrial pressure < 15 mmHg and PAPi ≥ 1.0 [62]; and if met, a final decision is made to wean from VA-ECMO. Since VA-ECMO reduces RV preload, preserved RV function is essential to wean from VA-ECMO. Although right atrial pressure and PAPi can be used to some extent to assess RV performance for transient changes even under VA-ECMO support, it should be noted that PAPi could be influenced by RV preload. The third step requires meeting the criteria of PAWP < 20 mmHg and CPO ≥ 0.6 watts [63]; and if met, a final decision is made to wean from Impella.

Impella is a percutaneous circulatory support device capable of LV venting and incremental aortic blood flow delivery [64]. Improvement of PAWP and CPO directly indicate hemodynamic effects induced by Impella implantation. Physician should ensure that these indicators do not worsen when the Impella P-level is reduced. Elevated PAWP during the off test has been reported to be associated with unsuccessful weaning in patients with implantable LV assist device [65]. Among echocardiographic parameters, increase of LV outflow tract velocity time integral (LVOT-VTI) has been reported to be an indicator of LV recovery and a criterion to decide weaning from Impella [66]. The final decision to decannulate should be made at minimum VA-ECMO and Impella flows (VA-ECMO: 1–2 L/min, usually 1.5 L/min; and Impella: P-level 2). If the criteria are not met at any stage, the weaning process should not proceed, and intensification of heart failure therapy or device escalation should be considered. The frequency of concomitant significant mitral regurgitation on echocardiography was reported to be higher in patients with higher PAWP or lower CPO at Impella weaning [63]. These patients who are at high risk of Impella weaning failure should be evaluated for severity of concomitant mitral regurgitation, and additional interventions such as surgical or edge-to-edge mitral valve repair may be considered prior to decannulation. There is a paucity of evidence focusing on t-MCS weaning which have been validated in clinical trials, and only expert consensus exists. Verification by large-scale cohort studies is necessary in the future.

## Risk scores for the management of cardiogenic shock

Various risk scores have been proposed for the management of cardiogenic shock. Kalra et al. [67] reviewed the cardiogenic shock risk scores by classifying the currently available risk scores into 3 categories: (1) those focused on critically ill patients requiring intensive care; (2) those focused on patients with AMI and cardiogenic shock; and (3) those pertinent to patients with cardiogenic shock who are being sustained by mechanical support devices. Regarding the risk scores for estimation of outcome of patients managed by t-MCS, IABP Shock II risk score [68], SAVE (Survival After Veno-arterial ECMO) score [69], and ENCOURAGE (prEdictioN of Cardiogenic shock OUtcome foR AMI patients salvaGed by VA-ECMO) mortality risk score [70] aim to stratify the risk of hemodynamic instability (Table 1). However, none of these measures are sufficient to comprehensively capture the pathophysiology of cardiogenic shock along the time axis based on recent trends such as the SCAI cardiogenic shock classification, and they also fail to incorporate parameters that may affect the outcome of new t-MCS platforms such as Impella. An ideal risk prediction model should balance the incorporation of key hemodynamic parameters while still allowing dynamic use in multiple scenarios from guiding early decision-making to device weaning.

**Table 1:** Risk scores for management of cardiogenic shock

Study	**Variables**	Score ranges
IABP shock II risk score [68]	6 indices: ① Age > 73 years (1 pt) ② History of stroke (2 pt) ③ BS > 10.6 mmol/L (191 mg/dL) (1 pt) ④ Cre > 132.6 μmol/L (1.5 mg/dL) (1 pt) ⑤ Arterial lac > 5 mmol/L (2 pt) ⑥ TIMI flow grade < 3 after PCI (2 pt)	30-day mortality risk Low (0–2) (24%) Intermediate (3–4) (49%) High (5–9) (77%)
SAVE score [69]	7 indices: ① Etiology: myocarditis (3 pt), refractory VT/VF (2 pt), post heart/lung transplantation (3 pt), congenital heart disease (− 3 pt) ② Age : 18–38 yr (7 pt), 39–52 yr (4 pt), 53–62 yr (3 pt), ≥ 63yr (0 pt) ③ Weight : < 65kg (1 pt), 65–89 kg (2 pt), ≥ 90 kg (0 pt) ④ Cardiac: pre-ECMO cardiac arrest (− 2 pt), diastolic BP before ECMO ≥ 40 mmHg (3 pt), pulse pressure before ECMO ≤ 20 mmHg (− 2 pt) ⑤ Respiratory: peak inspiratory pressure ≤ 20 cmH_2_O (3 pt), duration of intubation pre ECMO; ≤ 10 h (0 pt), 11–29 h (−2 pt), ≥ 30h (− 4 pt) ⑥ Renal dysfunction : acute renal failure (− 3 pt), chronic renal failure (− 6 pt), HCO_3_ pre ECMO ≤ 15 mmol/L (− 3 pt) ⑦ Other organ failure: liver failure (− 3 pt), CNS dysfunction (− 3 pt)	In-hospital survival rate Risk I >5 (75%) Risk II 1 to 5 (58%) Risk III − 4 to 0 (42%) Risk IV − 9 to − 5 (30%) Risk V ≤ − 10 ( (18%)
ENCOURAGE mortality risk score [70]	7 indices: ① Age >60 (5 pt) ② Gender: female (7 pt) ③ BMI > 25 kg/m^2^ (6 pt) ④ GCS < 6 (6 pt) ⑤ Cre >150 μmol/L or 1.7 mg/dL (5 pt) ⑥ Lac : < 2 mmol/L (0 pt), 2–8 mmol/L (8 pt), > 8 mmol/L (11 pt) ⑦ Reduced prothrombin activity < 50% (5 pt)	1 month survival rate 0–12 (92%) 13–18 (70%) 19–22 (35%) 23–27 (28%) ≥ 28 (17%)

*BS* blood sugar, *Cre* creatinine, *TIMI* thrombolysis in myocardial infarction, *ECMO* extra corporeal membrane oxygenation, *BP* blood pressure, *CNS* central nerve system, *BMI* body mass index, *Lac* lactate

This figure was synthesized from the articles on IABP Shock II risk score [68], SAVE (Survival After Veno-arterial ECMO) score [69] and ENCOURAGE (prEdictioN of Cardiogenic shock OUtcome foR AMI patients salvaGed by VA-ECMO) mortality risk score [70]

## Protocolized regional cardiogenic shock network and shock team management

Management of cardiogenic shock using t-MCS requires seamless treatment from pre-hospital to ER, in the catheter laboratory, and in the cardiovascular intensive care unit. It is essential that the treatment should be carried out efficiently with a coordinated multidisciplinary shock team approach. From this point of view, transfer of patients with refractory cardiogenic shock to centers with appropriate interdisciplinary expertise and adequate patient volume is recommended to ensure optimal outcomes, and community hospitals are encouraged to develop collaborative care models when local resources and expertise are not available [71]. Recent guidelines recommend that developing regional care system integrating MCS-capable hospitals (hubs) and spoke centers with defined protocols for early recognition, treatment, and transfer may improve outcomes of cardiogenic shock patients [72]. Additionally, the guidelines also recommend that acute MCS hospitals should be available to support spoke centers at all times. Potential benefits of standardized care for cardiogenic shock across regional care networks are reported from Maryland, USA [73]. In metropolitan Tokyo, an urban cardiovascular care unit network consisting of hospitals capable of performing emergency PCI at any time is in operation [74], but only approximately 40% of all the hospitals are Impella-certified. In order to improve the quality of care for cardiogenic shock patients in Tokyo, it seems necessary to build a special network applying standardized protocols, including transfer to MCS (including Impella)-capable hospitals. Future development should include building a cardiogenic shock network system with defined protocols based on the background and characteristics of each region.

Within individual hospital systems, adoption of an interdisciplinary shock team may further improve clinical outcomes [75, 76]. The goals of the shock team and collaboration with a centralized cardiogenic shock hub are to streamline care, minimize treatment delays, and centralize advanced heart failure services [77]. The shock team and centralized cardiogenic shock hub also ensure equitable access to high-quality care and opportunities to escalate to t-MCS where appropriate. Tehrani et al. [78] compared in-hospital mortality in patients with no compensated heart failure shock before and after the use of a cardiogenic shock team protocol and found a reduction in mortality from 40 to 28% and improvement in outcomes after using the shock team protocol. A cohort study (National Cardiogenic Shock Initiative) conducted in the United States from 2016 to 2019 at 35 centers also reported in-hospital mortality rate of 28% as a result of treating patients with AMI shock (AMI-cardiogenic shock) using a uniform shock team protocol [59]. This rate compares favorably to in-hospital mortality in the SHOCK trial (53% 1993–1997) [79], the IABP SHOCK trial (60%, 2009–2012) [13], and the CULPRIT-SHOCK trial (49%, 2013–2017) [80], which are previous large cohort studies of treatment of cardiogenic shock associated with AMI.

## Conclusion

The basic principle of treatment of patients with shock is to immediately identify the etiology and extent of the shock, maintain adequate tissue perfusion, and provide appropriate management. Improvement of the outcome of cardiogenic shock requires stabilization of hemodynamics and LV unloading using t-MCS based on early identification and staging of shock, in parallel with appropriate treatments of the underlying disease, such as PCI for early revascularization of coronary artery, as well as adjunctive intensive care management. In addition, for the treatment of cardiogenic shock using t-MCS devices, multidisciplinary shock protocols for heart failure management are required, in order not only to save the patient’s life, but also to preserve cardiac function, which may lead to heart recovery.

## Data Availability

Data sharing not applicable to this article as no data sets were generated or analyzed during the current study.
